# Road Traffic Noise at the Residence, Annoyance, and Cognitive Function in Elderly Women

**DOI:** 10.3390/ijerph16101790

**Published:** 2019-05-20

**Authors:** Kateryna B. Fuks, Claudia Wigmann, Hicran Altug, Tamara Schikowski

**Affiliations:** IUF-Leibniz Research Institute for Environmental Medicine, 40225 Düsseldorf, Germany; kateryna.fuks@iuf-duesseldorf.de (K.B.F.); claudia.wigmann@iuf-duesseldorf.de (C.W.); hicran.altug@iuf-duesseldorf.de (H.A.)

**Keywords:** road traffic noise, noise annoyance, cognitive impairment

## Abstract

The detrimental effects of traffic noise on cognition in children are well documented. Not much is known about the health effects in adults. We investigated the association of residential exposure to road traffic noise and annoyance due to road traffic noise with cognitive function in a cohort of 288 elderly women from the longitudinal Study on the influence of Air pollution on Lung function, Inflammation and Aging (SALIA) in Germany. Residential noise levels—weighted 24-h mean (L_DEN_) and nighttime noise (L_NIGHT_)—were modeled for the most exposed facade of dwellings and dichotomized at ≥50 dB(A). Traffic noise annoyance (day and night) was estimated by questionnaire. Cognitive function was assessed using the Consortium to Establish a Registry on Alzheimer’s Disease (CERAD-Plus) Neuropsychological Assessment Battery. The modeled noise levels were associated with impaired total cognition and the constructional praxis domain, independently of air pollution. Self-reported noise annoyance was associated with better performance in semantic memory and constructional praxis domains. This finding should be interpreted with caution since we could not control for potential confounding by hearing loss. Noise levels and annoyance were associated, but their health effects seemed mutually independent.

## 1. Introduction

Adverse health effects of ambient noise exposure are divided into auditory and non-auditory [1]. There is only one auditory effect—hearing loss. The non-auditory effects, suggested by epidemiologic and mechanistic studies, include cardiovascular diseases, annoyance, and sleep disturbance [1]. Noise has been shown to adversely affect cognitive function in children, causing communication difficulties, impaired attention, increased arousal, learned helplessness, frustration, annoyance, and sleep disturbance [1,2]. 

Not much is known about the effects of noise on cognition in adults. Elmenhorst and colleagues investigated the short-term effects of nocturnal air traffic noise on cognitive function in healthy volunteers [3]. The authors found that nighttime traffic noise exposure was associated with impaired reaction times [3,4]. Similarly, in a panel study on nighttime aircraft noise exposure, Schapkin and colleagues observed a selective impairment of the inhibitory functioning and worsening in sleep quality in some participants [5]. However, the results were mixed: the authors observed no decrement in performance after exposure, possibly due to a compensatory effect [5]. In another panel study, noise exposure was associated with slower psychomotor speed, reduced episodic and working memory, and more cautious decision making; factors like cognition, noise sensitivity, and sleep quality influenced these associations [6]. 

The long-term effects of residential noise exposure on cognition have been scarcely investigated so far, despite the dominance of this exposure in many populations. For example, about 125 million residents of the European Union are exposed to 24-h noise levels greater than 55 decibels [7]. It is plausible that acute impairments due to noise exposure could potentiate a chronic deterioration in cognitive function.

Only one study so far has investigated the chronic effect of road traffic noise on cognition [8]. Tzivian and colleagues studied the effects of traffic-related exposure to noise and air pollution on mild cognitive impairment (MCI) [8]. MCI is defined as a “cognitive decline greater than expected for an individual’s age and educational level, but that does not interfere notably with activities of daily life” and is a major risk factor for progression to Alzheimer’s disease ([9], p. 1262). Using data from a large population-based study, Tzivian and colleagues found positive associations of road traffic noise as well as exposure to fine particulate matter and soot with overall and amnestic, but not with non-amnestic, MCI [8]. The associations with road traffic noise were robust to adjustment for air pollution, but not vice versa [8].

In contrast to other environmental risks, such as air pollution or ionizing radiation, noise can be perceived by an affected person and cause annoyance, defined as “a feeling of resentment, displeasure, discomfort, dissatisfaction, or offense when noise interferes with someone’s thoughts, feelings, or actual activities” ([10], p. 126). Annoyance can lead to disturbed activities, somatic and psychosomatic health effects [10]. It is not clear yet whether annoyance can contribute to the effects of noise on cognitive function.

In this study, we aimed to broaden the evidence on the long-term effects of noise on cognition, using a cohort of elderly women from an industrialized area in Western Germany, including both modeled noise levels at the residence and self-estimated noise annoyance. 

## 2. Materials and Methods 

### 2.1. Study Population 

We used data from the Study on the influence of Air pollution on Lung function, Inflammation and Ageing (SALIA), an ongoing cohort study in the state of North Rhine-Westphalia in Germany. The design of the SALIA study has been described elsewhere [11,12,13]. Briefly, 4874 women aged 55 years took part in the baseline examination in 1985–1994. The study participants resided in five cities of the highly industrialized Ruhr area and in two counties in rural Münsterland with very low pollution levels, serving as a reference. The baseline response rate was 70% and was stable over time with no systematic differences between study areas [11]. In 2006–2007, a questionnaire on health status was sent to 4027 participants who were still alive and residing in the study area; 2116 responded (53%) and were invited to the clinical examination. In 2007–2010, all women who agreed to participate and whose lung function had been measured at baseline took part in a comprehensive clinical follow-up examination, comprising paper-assisted interview, cognitive assessment, lung function measurement, and blood test. The follow-up sample comprised 834 women (39% of the questionnaire follow-up sample and 17% of the baseline sample) aged 67 to 80 years. All participants gave written informed consent. The Medical Ethics Committee of the University of Bochum approved the follow-up examination (approval number 2732). 

### 2.2. Residential Noise Exposure

**Figure 1:**
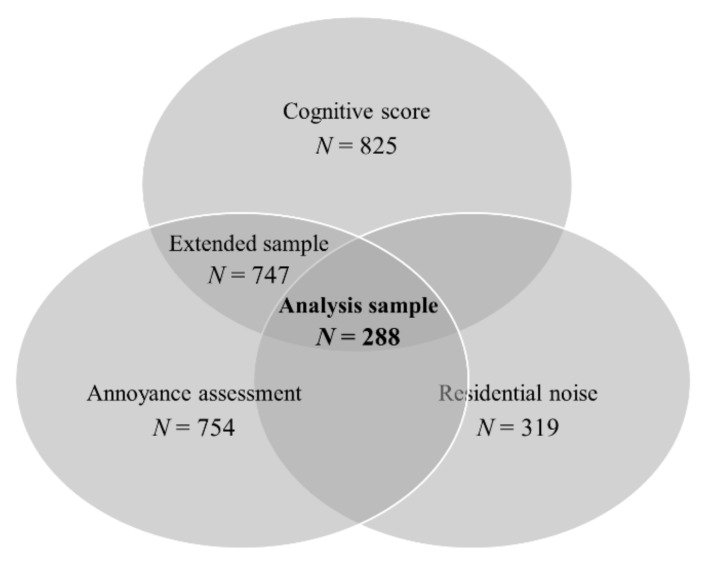
Numbers of participants with non-missing cognitive function and noise data.

We assessed modeled ambient road traffic noise as a measure of objective noise exposure for the year 2006 preceding the follow-up examination. Noise levels were modeled according to the EU Directive 2002/49/EC for the most exposed facade of dwellings [14]. Noise levels were modeled as weighted 24-h mean (L_DEN_) and night-time mean (L_NIGHT_). The modelled noise ranges were between 30.5–73.8 dB(A) and 21.7–63.5 dB(A) for 24-h mean and night-time mean road traffic noise levels, respectively. For the analyses, noise variables were dichotomized at ≥50 dB(A). Noise data were available only in a subset of participants, all residing in the Ruhr area (*N* = 319; Figure 1).

### 2.3. Noise Annoyance

Annoyance due to traffic noise (“noise annoyance”) was estimated by a 5-point scale recommended by the ICBEN (International Commission on Biological Effects of Noise) [15]. The study participants were asked: “How much do you generally feel disturbed or annoyed by traffic noise when being at home?”. Day- and night-time annoyances were assessed separately. The answers were assessed with a Likert scale as “not at all”, “somewhat”, “moderately”, “strongly”, and “very strongly”. We considered annoyance present for answers “somewhat”, “moderately”, “strongly”, and “very strongly”. The information on traffic noise annoyance was available for 754 participants (Figure 1).

### 2.4. Assessment of Cognitive Function

The assessment of cognitive function in the SALIA study has already been described [12,16]. Cognitive function was assessed using the Consortium to Establish a Registry on Alzheimer’s Disease (CERAD) Neuropsychological Assessment Battery. The German version of it, CERAD-Plus, was applied (Memory Clinic 2009). The cognitive tests were administered by trained personnel and double checked by a clinically trained psychologist. CERAD-Plus consists of 18 test items belonging to four cognitive domains and Mini-Mental State Examination (MMSE). All items were standardized for age and educational level as z-scores using reference values from a German-speaking population. A z-score of 0 and higher indicates cognitive performance equal to or higher than the one expected for the participant’s age and educational level, while a score < 0 indicates impaired cognition. Similarly to a previous study on the adverse effects of air pollution on cognitive scores in the SALIA cohort [12], we selected 10 items which assess tasks directly performed by the participant as well as MMSE and the total CERAD score (sum of all 18 z-scores) to assess the global cognition (Table 1). All z-scores were dichotomized as <0 and ≥0 (reference) for the analyses. Scores for at least one CERAD-Plus item were available for 825 participants (Figure 1).

### 2.5. Covariates

Information on smoking status and exposure to passive smoke was assessed via interview. Similar to the previous analyses with the CERAD-Plus score in SALIA [12], we additionally adjusted for socio-economic levels, assessed as the highest educational level of the participant or her spouse: low (<10 years), medium (10 years), and high (>10 years). As cognitive performance has been strongly linked to depression [17], we assessed diagnosed depression via self-report. As previous analyses with the SALIA study have shown an association of air pollution exposure with lower cognitive function [12,16], we included air pollution as a covariate in our analyses. Air pollution was assessed using the ESCAPE LUR model approach [18] as a yearly mean concentration of particulate matter with an aerodynamic diameter of ≤10 µm (PM_10_) and nitrogen dioxide (NO_2_) at the residence. 

### 2.6. Statistical Analyses

We used logistic regression to analyze the associations of residential noise exposure and annoyance due to road traffic noise with cognitive function (analysis sample *N* = 288, Figure 1). In the main model, we a priori adjusted for age (linear, squared, and cubic terms), smoking, passive smoking, and educational level. In an additional step, we adjusted for (1) annoyance in models with noise and noise in models with annoyance; (2) air pollutants PM_10_ and NO_2_; and (3) depression. Other sensitivity analyses included (1) employing the main model in the extended sample with non-missing subjective noise (*N* = 747), and (2) using different cutpoints for dichotomizing the annoyance (“moderately” instead of “somewhat”) and traffic noise levels (≥40 dB for L_NIGHT_). In addition, we performed an interaction analysis by combining objective and subjective noise levels in one exposure variable with levels “low/low”, “high/low”, “low/high”, and “high/high”. Analyses were performed with R version 3.5.0 [19].

## 3. Results

The analysis sample contained 288 participants with non-missing information on residential noise exposure, annoyance, and cognitive score (Figure 1 and Table 2). The mean age was about 75 years, almost half (46.9%) of the participants or their spouses had a medium educational status (Table 2). Almost none of the participants smoked (3.8% current smokers), while about two-thirds reported exposure to passive smoking at home (60.8%). Regarding depression, 11.8% of the participants reported physician-diagnosed depression. We observed no substantial differences between the analysis sample and the extended study sample (Supplementary Materials, Table S1).

Mean levels of PM_10_ and NO_2_ at the residence were 28.0 and 32.2 µg/m^3^, respectively. Mean residential noise levels were 47.2 dB(A) L_NIGHT_ and 55.9 dB(A) L_DEN_, with 74.7% and 35.4% of participants being exposed to noise above the selected cutpoint (Table 2), respectively. Fifty-six percent of participants reported no annoyance by traffic noise in the daytime, and 76.7% reported no annoyance by traffic noise at night. 

L_DEN_ and L_NIGHT_ were strongly correlated, with a Spearman’s rho of 0.99 (see Table 3), which is expected, since the definition of L_DEN_ embraces L_NIGHT_. Annoyance in the daytime and at night correlated moderately (0.63). We found weak correlations of noise annoyance with residential noise (0.24–0.35). While annoyance and residential noise were both weakly correlated with NO_2_ concentrations, the correlation of PM_10_ concentrations was stronger with residential noise levels than with noise annoyance (Table 3).

The average CERAD-Plus total score was below zero, with a mean value of −2.19 and a standard deviation of 10.10, and less than half of the study sample (44.9%) had a total score of ≥0 (Table 4). The distribution of the total score was left-skewed (Supplementary Materials, Figure S1). As for the individual tests, the average values were below 0 for 7 out of 11 tests, with 21.5% to 76.9% of participants having a score of ≥0 (Table 4).

### 3.1. Residential Noise Levels and Cognitive Function

**Figure 2:**
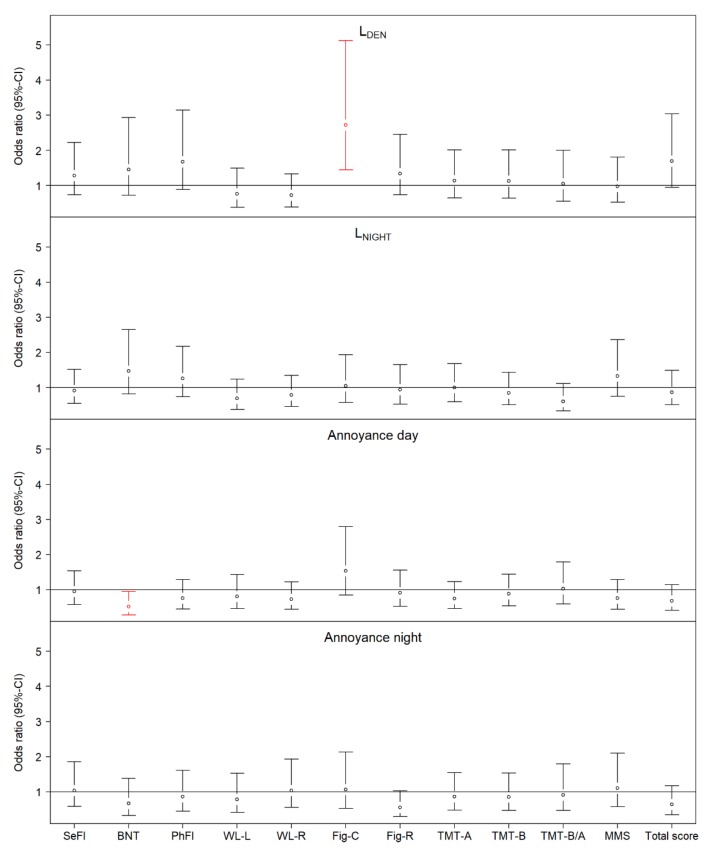
Association of residential noise and noise annoyance with dichotomized cognitive scores. Adjusted for age (linear, squared, and cubic terms), smoking, passive smoking, and educational level. We modeled the probability that score < 0 (cognitive performance lower than expected for the participant’s age and educational level). Statistically significant associations (*p* < 0.05) are marked red.

We observed no associations of L_NIGHT_ ≥ 50 dB(A) with cognitive scores in the main model (Figure 2 and Supplementary Materials, Table S2). However, using a lower cutpoint of ≥40 dB(A) yielded associations with an impaired score of the figure copying test, similar to the one observed with L_DEN_ (Supplementary Materials, Table S3). Adjusting for annoyance and air pollutants PM_10_ and NO_2_ revealed a borderline significant association (*p* < 0.1) of L_NIGHT_ ≥ 50 dB(A) with a better score on one of the trail making tests (Supplementary Materials, Table S2). 

### 3.2. Annoyance and Cognitive Function

As shown in Figure 2, annoyance at daytime was associated with a better performance on the Boston naming test from the semantic memory domain (Odds ratio (OR) 0.52; 95% CI: 0.28 to 0.95). Adjustment for residential noise levels and air pollutants, as well as analyses with the extended sample (*N* = 747), did not affect this association, but also revealed a borderline significant association with a better total score (Supplementary Materials, Tables S2 and S4). Moreover, in the extended sample, we observed a significant association with a better performance of word list learning from the episodic memory domain (Supplementary Materials, Table S4).

Annoyance at night showed a borderline significant relationship with better figure recall (constructional praxis): OR 0.56 (95% CI: 0.30 to 1.03). This estimate became statistically significant when adjusted for physician-diagnosed depression (Supplementary Materials, Table S2). In the model adjusted for air pollutants, we found a borderline significant (*p* < 0.1) association of nighttime annoyance by traffic noise with a better total CERAD-Plus score (Supplementary Materials, Table S2). When performing analyses with the extended dataset, we found no associations of nighttime annoyance with better cognitive function (Supplementary Materials, Table S4). Moreover, nighttime annoyance was linked to impaired semantic fluency test at *p* < 0.1 (Supplementary Materials, Table S4).

The findings with annoyance at day and night did not persist when using the alternative cutpoint “moderately” for the dichotomized annoyance definition (Supplementary Materials, Table S5). 

### 3.3. Combined Exposure to Noise and Annoyance with the Cognitive Function

When compared to participants who were not exposed to L_DEN_ ≥ 50 dB(A) and who also did not report annoyance by noise in the daytime (reference level), those who were exposed to high noise levels performed worse on figure copying, regardless of whether they reported annoyance or not (Supplementary Materials, Table S5). Participants with low noise exposure during nighttime but reporting annoyance showed better cognitive function according to the total CERAD score (Supplementary Materials, Table S6). 

### 3.4. Association of Noise with Annoyance

A logistic regression of objective noise levels on subjective noise annoyance revealed significant positive associations in the crude as well as in the main adjusted model (Supplementary Materials, Table S7). Residing at L_DEN_ levels of ≥50 dB(A) was associated with OR for annoyance at day of 2.38 (95% CI: 1.33 to 4.26); at L_NIGHT_ levels of ≥50 dB(A), the probability of nighttime annoyance was even higher (OR 3.21; 95% CI: 1.79 to 5.76). 

## 4. Discussion

We found complex relationships of noise exposure and noise annoyance with the cognitive performance of elderly women. While the ambient noise levels at the residence were associated with impaired cognitive performance in the constructional praxis domain (figure copying test) and with the total CERAD-Plus score, noise annoyance exhibited inverse associations with the domains of semantic memory (Boston naming test) and constructional praxis (figure recall). This is, to our knowledge, the first study on the long-term association of road traffic noise with cognition in the elderly that assessed noise annoyance in addition to ambient noise levels. However, this is an exploratory study and, due to the absent information on hearing loss, the results with annoyance might be biased. One should be cautious not to over-interpret the study findings.

The findings with residential noise levels were very similar to our earlier analysis of the SALIA cohort in which the effects of air pollution were investigated [12]. Previously, we reported a positive association of the long-term concentrations of particulate matter and nitrogen oxides at the residence with the impaired constructional praxis domain [12]. Similarities in the effects on cognition by noise and air pollution have been reported in another cohort study as well; the results with noise were more robust to adjustment for air pollution than the results with air pollution to adjustment with noise [8]. Our results with noise were independent of air pollution, as shown in the sensitivity analyses. 

Constructional praxis tests are indicators of visual-spatial deficits [12]. Poor scores in figure copying tasks have been observed in patients with Alzheimer’s disease (AD) or vascular dementia [20]. Constructional praxis copy scores allow for differentiating the advanced stages of AD or to discriminate between AD and depression; they were also useful in identifying brain pathology or the relationship between nutrition and cognitive function [21]. 

We found a somewhat counterintuitive positive correlation of annoyance and cognition: better scores in the semantic memory domain (Boston naming test) and in the constructional praxis domain (figure recall) were associated with higher annoyance of traffic noise in the daytime. In children, noise annoyance is correlated with lower cognition [22]. In older adults, annoyance might indicate a better hearing ability and thus better cognition; however, there are currently no studies available to confirm or refute this relationship. It is not likely that poor cognition drives poor perception directly, but rather that the relationship is quite complex, and one of the reasons for a worse cognition is a compensation to impaired perception [23]. In a panel study on noise, annoyance, and cognitive performance by Sandrock and colleagues, noisy conditions did not affect cognitive performance and mental strain in general, but a subgroup of participants with high noise sensitivity was more annoyed and performed worse [24]. We consider it possible that the results with annoyance were affected by an uncontrolled confounding factor due to the missing information on hearing loss. Hearing loss can affect both annoyance and cognition, fulfilling the definition of a confounder. Not adjusting for it might have introduced a spurious association between annoyance and cognition. Such confounding would likely drive the association away from null in a positive direction: if hearing loss is related to lower annoyance and lower cognition, than not adjusting for it would result in a spurious association of higher annoyance with higher cognition. Since we cannot control for hearing loss, the results with annoyance and cognition should be interpreted with caution.

As for the analyses with traffic noise, hearing loss is unlikely a confounder. It cannot affect the exposure and is highly unlikely a consequence of traffic noise exposure, considering the relatively low residential noise levels in the study population. 

We found a positive association of noise with annoyance, independent of potential confounders. This is in line with a known monotonic relationship of noise levels with annoyance [4]. However, the associations of noise and annoyance with cognitive scores were mutually independent. This observation is plausible. Although annoyance is a consequence of noise exposure, there are a number of factors that influence noise-related annoyance and could therefore contribute to the associations with cognition. Personal characteristics—for example, anxiety, fear of noise source, and feeling that exposure could be avoided—were shown to modify the relationship between noise exposure and annoyance [10]. Residential factors, such as type of housing, location of rooms, noise barriers, window opening habits, etc., could also influence the degree of annoyance in relation to noise [25]. Incorporating these factors in further studies could explain the complex relationships between noise exposure, annoyance, and cognitive function in the elderly. 

One of the theories aiming to explain noise effects on cognition is based on resource allocation theory [26]. Subjects, who were continuously exposed to noise (traffic police officers), demonstrated better discrimination and selection ability in the absence of noise, compared to the control subjects. This observation, according to Chiovenda and colleagues, reflects “their continuous need for diverting themselves from the environmental noise” ([26], p. 235). In the presence of noise, the difference in performance disappeared [26]. Noise-exposed subjects demonstrated higher susceptibility to background noise, reflected in a stronger regression of cognitive performance in the presence of background noise, than the controls [26]. In accordance with the resource allocation theory, the working memory network was modulated by exposure to background acoustic noise (reviewed in [27]). Noise can affect cognitive function through altered hippocampal signaling, as suggested by animal studies (reviewed in [8]). In an animal study, day- and nighttime traffic noise exposure caused the hypothalamic-pituitary-adrenal (HPA) axis hyperactivity, anxiety behavior, worse learning, memory, and coordination; it also affected the brain measures [28]. Noise-triggered activation of the HPA axis, measured as the concentration of its product cortisol, was observed in a panel study with healthy male volunteers [29]. The HPA axis coordinates the stress response, hippocampus, and memory [30]. Alterations of the HPA axis and hippocampus dysfunctions correlate with mental disorders, such as depression [30]. The hippocampus plays a role in spatial learning and memory [8]. There are many modulators of noise-related stress responses: for example, the feeling of control over the stressor affects the hormonal reaction to stress [31]. In addition, it is very probable that sleep disturbance due to noise affects cognitive function. Road traffic noise above 45 dB(A) can lead to sleep disturbance, if windows are opened [32]. Sleep duration and quality affect cognitive performance [33]. 

The limitations of the current study include possible exposure misclassification of traffic noise exposure, since noise exposure was approximated with a model, and no information was available on the modifying residential factors, such as ventilation habits. The study sample with available noise values was also quite small, thus diminishing the statistical power to detect associations. In addition, noise annoyance was measured via a rather crude scale. In a sensitivity analysis with another cutpoint we observed different results, thus non-linear relationships of annoyance with cognition are possible. As for the annoyance, a major limitation and possible source of bias is the lack of information on hearing loss, as discussed above. Moreover, due to the small number of participants reporting substantial annoyance, we used a lower cutpoint (“somewhat” in the main analyses and “moderately” in the sensitivity analyses). Such dichotomization sets a lower cutpoint than the one recommended by the International Commission on Biological Effects of Noise, which limits the comparability of our results. Using a conservative cutpoint does not allow us to differentiate between participants with only minor annoyance and those considerably annoyed. However, considering the small sample size of study participants with modeled traffic noise exposure, the selection of a lower cutpoint was necessary to avoid a statistical power problem. As in many longitudinal studies with elderly participants, healthy survivor bias could influence our results.

Our study has a number of strengths. We assessed day- and nighttime noise exposure simultaneously, and, in addition to modeled residential noise levels, were able to investigate the noise annoyance. Other strengths of the study include assessment of both noise and air pollution, and the validated cognition assessment, standardized for age and educational status. 

## 5. Conclusions

Our results reflect the complexity of the relationships of residential noise exposure and annoyance with cognitive performance. Residential noise levels were associated with impaired total cognition and the constructional praxis domain, independently of air pollution. Self-reported traffic noise annoyance was associated with better performance in semantic memory and the constructional praxis domain, which might be a spurious finding due to an uncontrolled confounding by hearing loss. The associations of modeled noise levels and self-reported annoyance with cognition were mutually independent.

## Figures and Tables

**Table 1:** The composition of the Consortium to Establish a Registry on Alzheimer’s Disease (CERAD)-Plus score.

Cognitive Domain	Subtests/Scores
Semantic memory	Semantic fluency test (SeFl), Phonetic fluency test/S words (PhFl), Boston naming test (BNT)
Episodic memory	Word list learning test (WL-L; sum of word list learning (1) ^1^ word list learning, (2) word list learning, (3) word list recall test (WL-R), word list intrusions, word list savings, word list recognition
Constructional praxis	Figure copying (Fig-C), figure recall (Fig-R), figure savings
Executive function	Trail making test (TMT-A, TMT-B, ratio TMT-B/A)
Mini-Mental State Examination (MMSE)	
Total score	

^1^ The scores highlighted in grey are only included in the total CERAD-Plus score and have not been analyzed separately since they are already included in other scores or do not represent tasks directly performed by the participant.

**Table 2:** Individual and residential characteristics of the analysis sample.

Variable		Statistics	*N*
*Individual characteristics*			
Age (years), mean ± SD		74.5 ± 2.2	288
Educational status, % ^1^	Low	16.70%	48
	Middle	46.90%	135
	High	36.10%	104
	Missing	0.30%	1
Smoking, %	Yes	3.80%	11
	No	96.20%	277
	Missing	0%	0
Passive smoking at home, %	Yes	60.80%	175
	No	38.50%	111
	Missing	0.70%	2
Physician-diagnosed depression	Yes	11.80%	34
	No	87.80%	253
	Missing	0.30%	1
*Residential characteristics*			
Residential noise			
L_DEN_ (dB(A)), mean ± SD		55.9 ± 7.7	288
L_DEN_ ≥ 50 dB(A), %		74.70%	215
L_NIGHT_ (dB(A)), mean ± SD		47.2 ± 7.4	288
L_NIGHT_ ≥ 50 dB(A), %		35.40%	102
Traffic noise annoyance			
Daytime, %	Not at all	55.90%	161
	Somewhat	18.80%	54
	Moderate	14.60%	42
	Strong	7.60%	22
	Very strong	3.10%	9
	Missing	0%	0
Night, %	Not at all	76.70%	221
	Somewhat	11.10%	32
	Moderate	6.20%	18
	Strong	3.10%	9
	Very strong	2.80%	8
	Missing	0%	0
Air pollution			
PM_10_ (µg/m^3^), mean ± SD		28.0 ± 2.3	288
NO_2_ (µg/m^3^), mean ± SD		32.2 ± 7.4	288

^1^ The highest educational status of the participant or her spouse.

**Table 3:** Spearman correlations of the residential characteristics in the analysis sample (*N* = 288).

Parameter	Annoyance Night	L_DEN_	L_NIGHT_	NO_2_	PM_10_
Annoyance day	0.63	0.35	0.34	0.32	0.16
Annoyance night	1	0.24	0.24	0.28	0.17
L_DEN_		1	0.99	0.34	0.41
L_NIGHT_			1	0.32	0.40
NO_2_				1	0.49
PM_10_					1

**Table 4:** Description of the CERAD-Plus scores in the study sample.

Test	Subtest	Mean	Standard Deviation	Score ≥ 0 (%)
Semantic memory	SeFl	−0.09	0.91	46.9% (*N* = 288)
	BNT	0.76	1.07	76.9% (*N* = 286)
	PhFl	0.63	1.13	69.7% (*N* = 284)
Episodic memory	WL-L	−0.74	1.19	26.6% (*N* = 282)
	WL-R	−0.19	1.14	37.7% (*N* = 281)
Constructional praxis	Fig-C	−0.76	1.37	21.5% (*N* = 284)
	Fig-R	−0.53	1.11	28.1% (*N* = 281)
Executive function	TMT-A	−0.11	1.00	42.3% (*N* = 284)
	TMT-B	0.39	1.02	62.0% (*N* = 284)
	TMT-B/A	0.55	0.95	73.5% (*N* = 283)
MMSE		−0.82	1.25	27.1% (*N* = 288)
Total score		−2.19	10.10	44.9% (*N* = 276)

## Supplementary Materials

The following are available online at https://www.mdpi.com/1660-4601/16/10/1790/s1: Table S1. Description of the extended sample (*N* = 747); Table S2: Adjusted associations of residential noise levels and annoyance with dichotomized cognitive scores (*N* = 288). Results are presented as odds ratios with 95% confidence intervals; Table S3. Adjusted association of LNIGHT ≥ 40 dB(A) with dichotomized cognitive scores; Table S4. Adjusted association of noise annoyance with dichotomized cognitive scores in the extended dataset (*N* = 747); Table S5. Adjusted association of annoyance (cutpoint “moderately”) with dichotomized cognitive scores; Table S6. Effect modification analysis, using combined residential noise exposure and annoyance at night in the main adjusted model. Odds ratios and 95% confidence intervals are presented; Table S7. Associations of residential noise with annoyance; Figure S1. Distributions of CERAD-Plus z-scores in main sample (*N* = 288).
