# Supplementary material for: Road Traffic Noise at the Residence, Annoyance, and Cognitive Function in Elderly Women
Source: Int J Environ Res Public Health. 2019 May 20;16(10):1790. doi: 10.3390/ijerph16101790 (PMC6572214; doi:10.3390/ijerph16101790)
Supplement: Supplementary file 1 [file ijerph-16-01790-s001.pdf]

Supplementary Material

Throughout this Supplementary Material statistical significance ($p < 0.05$) is indicated by boldface while borderline significance ($0.05 < p < 0.1$) is indicated by italics.

Table S1. Description of the extended sample [1] (*N* = 747).

Variable	Statistics	*N*
Age (years), mean ± SD	73.4 ± 3.1	747
Educational status (%) [2]:		
Low	16.9%	126
Middle	47.9%	358
High	35.1%	262
Missing	0.1%	1
Smoking (%)		
Yes	2.9%	22
No	97.1%	725
Missing	0%	0
Passive smoking at home (%)		
Yes	60.0%	448
No	39.4%	294
Missing	0.7%	5
Physician-diagnosed depression (%)		
Yes	11.0%	82
No	88.6%	662
Missing	0.4%	3

[1] Study population with non-missing annoyance information and cognitive score (see Figure 1). [2] The highest educational status of the participant or her spouse.

Table S2. Adjusted associations of residential noise levels and annoyance with dichotomized cognitive scores [1] (*N* = 288). Results are presented as odds ratios with 95% confidence intervals.

Cognitive Score	Model	Residential Noise		Noise Annoyance	
		L_{DEN}	L_{NIGHT}	Day	Night
SeFl	Main [2]	1.28 (0.73, 2.22)	0.91 (0.55, 1.52)	0.95 (0.58, 1.54)	1.04 (0.59, 1.85)
	+noise [3]	1.29 (0.73, 2.26)	0.91 (0.54, 1.54)	0.91 (0.56, 1.49)	1.07 (0.59, 1.93)
	+PM_{10}, NO_2 [4]	1.49 (0.82, 2.71)	1.01 (0.59, 1.74)	1.01 (0.61, 1.67)	1.13 (0.62, 2.04)
	+depression [5]	1.28 (0.73, 2.22)	0.91 (0.55, 1.52)	0.95 (0.58, 1.54)	1.04 (0.59, 1.86)
BNT	Main	1.45 (0.72, 2.93)	1.47 (0.82, 2.65)	**0.52 (0.28, 0.95)** *,[6]	0.67 (0.33, 1.38)
	+noise	1.56 (0.77, 3.20)	1.55 (0.85, 2.84)	**0.48 (0.26, 0.88)** *	0.59 (0.28, 1.23)
	+PM_{10}, NO_2	1.08 (0.51, 2.29)	1.16 (0.62, 2.17)	**0.41 (0.21, 0.78)** **	0.53 (0.25, 1.14)
	+depression	1.45 (0.72, 2.93)	1.46 (0.81, 2.64)	**0.51 (0.28, 0.94)** *	0.66 (0.32, 1.35)
PhFl	Main	1.67 (0.89, 3.14)	1.26 (0.74, 2.17)	0.76 (0.45, 1.29)	0.86 (0.45, 1.61)
	+noise	1.88 (0.99, 3.58)	1.37 (0.78, 2.39)	0.70 (0.41, 1.19)	0.79 (0.41, 1.52)
	+PM_{10}, NO_2	1.64 (0.84, 3.20)	1.19 (0.67, 2.13)	0.71 (0.41, 1.22)	0.79 (0.41, 1.53)
	+depression	1.67 (0.89, 3.14)	1.26 (0.73, 2.17)	0.75 (0.44, 1.28)	0.84 (0.45, 1.59)
WL-L	Main	0.76 (0.38, 1.49)	0.69 (0.38, 1.24)	0.81 (0.46, 1.43)	0.79 (0.41, 1.53)
	+noise	0.76 (0.38, 1.52)	0.72 (0.39, 1.34)	0.84 (0.47, 1.50)	0.88 (0.44, 1.75)
	+PM_{10}, NO_2	0.81 (0.40, 1.67)	0.67 (0.36, 1.24)	0.77 (0.42, 1.38)	0.73 (0.37, 1.45)
	+depression	0.75 (0.38, 1.49)	0.68 (0.38, 1.23)	0.79 (0.45, 1.40)	0.76 (0.39, 1.48)
WL-R	Main	0.72 (0.39, 1.33)	0.79 (0.46, 1.35)	0.73 (0.44, 1.22)	1.04 (0.56, 1.93)
	+noise	0.76 (0.41, 1.40)	0.80 (0.46, 1.38)	0.76 (0.45, 1.28)	1.12 (0.59, 2.13)
	+PM_{10}, NO_2	0.70 (0.36, 1.33)	0.74 (0.42, 1.31)	0.67 (0.39, 1.15)	0.96 (0.51, 1.84)
	+depression	0.72 (0.39, 1.33)	0.79 (0.46, 1.36)	0.74 (0.44, 1.23)	1.05 (0.56, 1.97)
Fig-C	Main	**2.72 (1.44, 5.12)** **	1.05 (0.58, 1.93)	1.54 (0.85, 2.80)	1.07 (0.53, 2.13)
	+noise	**2.74 (1.43, 5.24)** *	1.06 (0.57, 1.97)	1.32 (0.71, 2.43)	1.05 (0.52, 2.15)
	+PM_{10}, NO_2	**2.54 (1.28, 5.03)** **	0.81 (0.42, 1.55)	1.31 (0.71, 2.43)	0.85 (0.41, 1.75)
	+depression	**2.72 (1.44, 5.12)** **	1.05 (0.57, 1.92)	1.54 (0.85, 2.80)	1.06 (0.53, 2.12)
Fig-R	Main	1.34 (0.73, 2.45)	0.94 (0.53, 1.65)	0.91 (0.53, 1.56)	*0.56 (0.30, 1.03)* [(*)]
	+noise	1.41 (0.76, 2.63)	1.03 (0.57, 1.86)	0.86 (0.50, 1.49)	*0.54 (0.28, 1.03)* [(*)]

Cognitive Score	Model	Residential Noise		Noise Annoyance	
		LDEN	LNIGHT	Day	Night
	+PM_{10}, NO_2	1.53 (0.79, 2.94)	0.97 (0.53, 1.79)	0.91 (0.52, 1.59)	*0.53 (0.28, 1.01)* (*)
	+depression	1.34 (0.73, 2.46)	0.93 (0.53, 1.64)	0.89 (0.52, 1.53)	**0.53 (0.28, 1.00)** *
TMT-A	Main	1.14 (0.65, 2.01)	1.00 (0.60, 1.68)	0.75 (0.46, 1.23)	0.86 (0.48, 1.55)
	+noise	1.23 (0.69, 2.20)	1.06 (0.62, 1.81)	0.72 (0.44, 1.20)	0.85 (0.46, 1.56)
	+PM_{10}, NO_2	1.17 (0.64, 2.15)	0.96 (0.55, 1.67)	0.68 (0.41, 1.14)	0.79 (0.43, 1.45)
	+depression	1.15 (0.65, 2.03)	0.98 (0.58, 1.66)	0.70 (0.43, 1.16)	0.78 (0.42, 1.42)
TMT-B	Main	1.13 (0.64, 2.01)	0.85 (0.51, 1.43)	0.88 (0.54, 1.44)	0.85 (0.47, 1.54)
	+noise	1.14 (0.64, 2.05)	0.83 (0.48, 1.41)	0.86 (0.52, 1.42)	0.88 (0.47, 1.63)
	+PM_{10}, NO_2	1.22 (0.66, 2.25)	0.86 (0.49, 1.49)	0.87 (0.52, 1.46)	0.84 (0.45, 1.56)
	+depression	1.12 (0.63, 1.98)	0.84 (0.50, 1.41)	0.84 (0.51, 1.38)	0.79 (0.43, 1.45)
TMT-B/A	Main	1.05 (0.55, 2.00)	0.61 (0.34, 1.12)	1.03 (0.60, 1.79)	0.91 (0.47, 1.80)
	+noise	1.07 (0.55, 2.07)	*0.59 (0.32, 1.10)* (*)	1.03 (0.59, 1.79)	1.08 (0.53, 2.18)
	+PM_{10}, NO_2	1.00 (0.50, 1.99)	*0.56 (0.29, 1.07)* (*)	1.06 (0.60, 1.87)	0.93 (0.46, 1.88)
	+depression	1.04 (0.55, 1.99)	0.61 (0.33, 1.11)	1.02 (0.59, 1.77)	0.89 (0.45, 1.76)
MMS	Main	0.97 (0.52, 1.81)	1.33 (0.75, 2.36)	0.76 (0.44, 1.29)	1.10 (0.58, 2.10)
	+noise	0.98 (0.52, 1.85)	1.38 (0.76, 2.49)	0.75 (0.44, 1.30)	1.01 (0.52, 1.98)
	+PM_{10}, NO_2	0.85 (0.44, 1.65)	1.26 (0.68, 2.34)	0.72 (0.42, 1.26)	1.08 (0.55, 2.10)
	+depression	0.97 (0.52, 1.81)	1.33 (0.75, 2.36)	0.75 (0.44, 1.28)	1.08 (0.57, 2.07)
Total score	Main	*1.69 (0.94, 3.04)* (*)	0.87 (0.51, 1.49)	0.68 (0.41, 1.14)	0.64 (0.35, 1.17)
	+noise	**1.84 (1.01, 3.38)** *	0.95 (0.54, 1.65)	*0.62 (0.36, 1.04)* (*)	0.65 (0.35, 1.21)
	+PM_{10}, NO_2	*1.87 (0.99, 3.52)* (*)	0.83 (0.47, 1.48)	*0.63 (0.37, 1.07)* (*)	*0.58 (0.31, 1.09)* (*)
	+depression	*1.68 (0.93, 3.04)* (*)	0.87 (0.51, 1.49)	0.67 (0.40, 1.13)	0.63 (0.34, 1.16)

[1] We modeled the probability that score < 0 (cognitive performance lower than expected for the participant's age and educational level).
[2] Adjusted for age (linear, squared, and cubic terms), smoking, passive smoking, and educational level.
[3] The models with LDEN and LNIGHT as main exposure were adjusted for annoyance (day or night, respectively). The models with annoyance as main exposure were adjusted for LDEN or LNIGHT, correspondingly.
[4] Main model additionally adjusted for PM_{10} and NO_2.
[5] Main model additionally adjusted for physician-diagnosed depression.
[6] Statistical significance: (*) $p < 0.1$, * $p < 0.05$, ** $p < 0.01$.
Statistical significance (p < 0.05) is indicated by boldface while borderline significance (0.05 < p < 0.1) is indicated by italics.

Table S3. Adjusted [1] association of $L_{NIGHT} \geq 40$ dB(A) with dichotomized cognitive scores [2].

Cognitive Score	Odds Ratio (95% CI)	*p*-Value
SeFl	1.04 (0.54, 2.03)	0.8971
BNT	1.51 (0.64, 3.54)	0.3453
PhFl	1.38 (0.66, 2.89)	0.3890
WL-L	0.89 (0.39, 2.05)	0.7895
WL-R	1.05 (0.51, 2.18)	0.8966
Fig-C	**2.27 (1.09, 4.72)**	**0.0285**
Fig-R	0.98 (0.46, 2.09)	0.9630
TMT-A	1.10 (0.55, 2.20)	0.7824
TMT-B	0.64 (0.32, 1.26)	0.1937
TMT-B/A	0.98 (0.44, 2.16)	0.9555
MMS	0.91 (0.42, 1.95)	0.8021
Total score	1.74 (0.85, 3.55)	0.1289

[1] Adjusted for age (linear, squared, and cubic terms), smoking, passive smoking, and educational level. [2] We modeled the probability that score < 0 (cognitive performance lower than expected for the participant's age and educational level). Statistical significance (p < 0.05) is indicated by boldface while borderline significance (0.05 < p < 0.1) is indicated by italics.

Table S4. Adjusted [1] association of noise annoyance with dichotomized cognitive scores [2] in the extended dataset (*N* = 747).

Cognitive Score	Annoyance at Day		Annoyance at Night	
	Odds Ratio (95% CI)	*p*-Value	Odds Ratio (95% CI)	*p*-Value
SeFl	1.18 (0.87, 1.6)	0.2833	*1.42 (0.96, 2.1)*	*0.0772*
BNT	**0.67 (0.48, 0.95)**	**0.0247**	0.69 (0.44, 1.08)	0.1046
PhFl	0.86 (0.62, 1.19)	0.3641	0.86 (0.57, 1.32)	0.5003
WL-L	0.79 (0.55, 1.12)	0.1853	0.84 (0.54, 1.3)	0.4266
WL-R	**0.69 (0.5, 0.95)**	**0.0238**	0.91 (0.6, 1.36)	0.6304
Fig-C	1.16 (0.78, 1.72)	0.4608	0.99 (0.6, 1.64)	0.9838
Fig-R	0.84 (0.59, 1.21)	0.3478	0.70 (0.45, 1.08)	0.1071
TMT-A	0.91 (0.66, 1.24)	0.5490	0.84 (0.56, 1.24)	0.3744
TMT-B	0.83 (0.61, 1.14)	0.2465	0.82 (0.55, 1.23)	0.3417
TMT-B/A	0.93 (0.66, 1.3)	0.6597	0.77 (0.49, 1.2)	0.2488
MMS	1.09 (0.77, 1.54)	0.6379	1.13 (0.72, 1.76)	0.5974
Total score	*0.75 (0.54, 1.04)*	*0.0817*	0.79 (0.53, 1.18)	0.2490

[1] Adjusted for age (linear, squared, and cubic terms), smoking, passive smoking, and educational level. [2] We modeled the probability that score < 0 (cognitive performance lower than expected for the participant's age and educational level). Statistical significance (p < 0.05) is indicated by boldface while borderline significance (0.05 < p < 0.1) is indicated by italics.

Table S5. Adjusted [1] association of annoyance (cutpoint "moderately") with dichotomized cognitive scores [2].

Cognitive Score	Annoyance at Day		Annoyance at Night	
	Odds Ratio (95% CI)	*p*-Value	Odds Ratio (95% CI)	*p*-Value

SeFl	0.93 (0.53, 1.61)	0.7915	1.09 (0.52, 2.29)	0.8151
BNT	0.96 (0.50, 1.83)	0.8935	0.97 (0.40, 2.33)	0.9445
PhFl	0.67 (0.36, 1.24)	0.2003	0.81 (0.35, 1.84)	0.6119
WL-L	1.23 (0.63, 2.41)	0.5390	0.68 (0.29, 1.55)	0.3557
WL-R	0.79 (0.44, 1.42)	0.4347	0.98 (0.44, 2.18)	0.9648
Fig-C	1.21 (0.61, 2.38)	0.5861	1.45 (0.56, 3.72)	0.4451
Fig-R	1.02 (0.54, 1.90)	0.9547	1.07 (0.46, 2.46)	0.8818
TMT-A	0.79 (0.45, 1.39)	0.4178	0.77 (0.36, 1.63)	0.4963
TMT-B	0.92 (0.52, 1.62)	0.7765	1.52 (0.72, 3.21)	0.2736
TMT-B/A	0.93 (0.49, 1.76)	0.8154	1.17 (0.50, 2.70)	0.7186
MMS	0.75 (0.41, 1.37)	0.3457	1.28 (0.55, 2.99)	0.5708
Total score	0.95 (0.53, 1.69)	0.8487	0.94 (0.43, 2.05)	0.8813

[1] Adjusted for age (linear, squared, and cubic terms), smoking, passive smoking, and educational level. [2] We modeled the probability that score < 0 (cognitive performance lower than expected for the participant's age and educational level).

Table S6. Effect modification analysis, using combined residential noise exposure and annoyance at night in the main adjusted model [1]. Odds ratios and 95% confidence intervals are presented.

**Cognitive Score [2]**	**L_{NIGHT} < 50 dB(A), No Annoyance at Night**	**L_{NIGHT} ≥ 50 dB(A), No Annoyance at Night**	**L_{NIGHT} < 50 dB(A), Annoyance at Night**	**L_{NIGHT} ≥ 50 dB(A), Annoyance at Night**
SeFl	1.00 (reference)	0.94 (0.51, 1.73)	1.16 (0.50, 2.70)	0.93 (0.44, 1.94)
BNT	1.00 (reference)	1.65 (0.84, 3.25)	0.59 (0.19, 1.89)	0.96 (0.39, 2.35)
PhFl	1.00 (reference)	1.64 (0.87, 3.09)	1.23 (0.50, 3.03)	0.85 (0.36, 1.98)
WL-L	1.00 (reference)	0.69 (0.34, 1.41)	0.84 (0.31, 2.31)	0.63 (0.27, 1.46)
WL-R	1.00 (reference)	0.68 (0.36, 1.29)	0.87 (0.35, 2.16)	0.97 (0.43, 2.16)
Fig-C	1.00 (reference)	1.16 (0.56, 2.41)	1.32 (0.45, 3.87)	1.00 (0.42, 2.37)
Fig-R	1.00 (reference)	1.33 (0.63, 2.78)	0.73 (0.29, 1.84)	0.52 (0.24, 1.14)
TMT-A	1.00 (reference)	1.29 (0.69, 2.43)	1.30 (0.53, 3.18)	0.74 (0.35, 1.57)
TMT-B	1.00 (reference)	1.11 (0.60, 2.04)	1.40 (0.59, 3.3)	0.60 (0.27, 1.36)
TMT-B/A	1.00 (reference)	0.61 (0.30, 1.26)	1.12 (0.42, 2.94)	0.63 (0.26, 1.53)
MMS	1.00 (reference)	1.00 (0.51, 1.98)	0.62 (0.26, 1.51)	1.80 (0.72, 4.47)
Total score	1.00 (reference)	0.70 (0.36, 1.35)	**0.37 (0.15, 0.89)** *,[3]	0.81 (0.37, 1.77)

[1] Adjusted for age (linear, squared, and cubic terms), smoking, passive smoking, and educational level. [2] We modeled the probability that score < 0 (cognitive performance lower than expected for the participant's age and educational level). [3] Statistical significance: (*) $p < 0.1$, * $p < 0.05$, ** $p < 0.01$. Statistical significance (p < 0.05) is indicated by boldface while borderline significance (0.05 < p < 0.1) is indicated by italics.

Table S7. Associations of residential noise with annoyance [1].

**Outcome**	**Crude Model**		**Main Model [2]**	
	**OR (95% CI)**	***p*-Value**	**OR (95% CI)**	***p*-Value**
Annoyance at day [3]	**2.21 (1.26, 3.9)**	**0.006**	**2.38 (1.33, 4.26)**	**0.0033**
Annoyance at night [4]	**3.29 (1.87, 5.78)**	**<0.0001**	**3.21 (1.79, 5.76)**	**0.0001**

[1] Dichotomized at "somewhat". [2] Adjusted for age (linear, squared, and cubic terms), smoking, passive smoking, and educational level. [3] Associations with LDEN. [4] Associations with LNIGHT. Statistical significance ($p < 0.05$) is indicated by boldface while borderline significance ($0.05 < p < 0.1$) is indicated by italics.

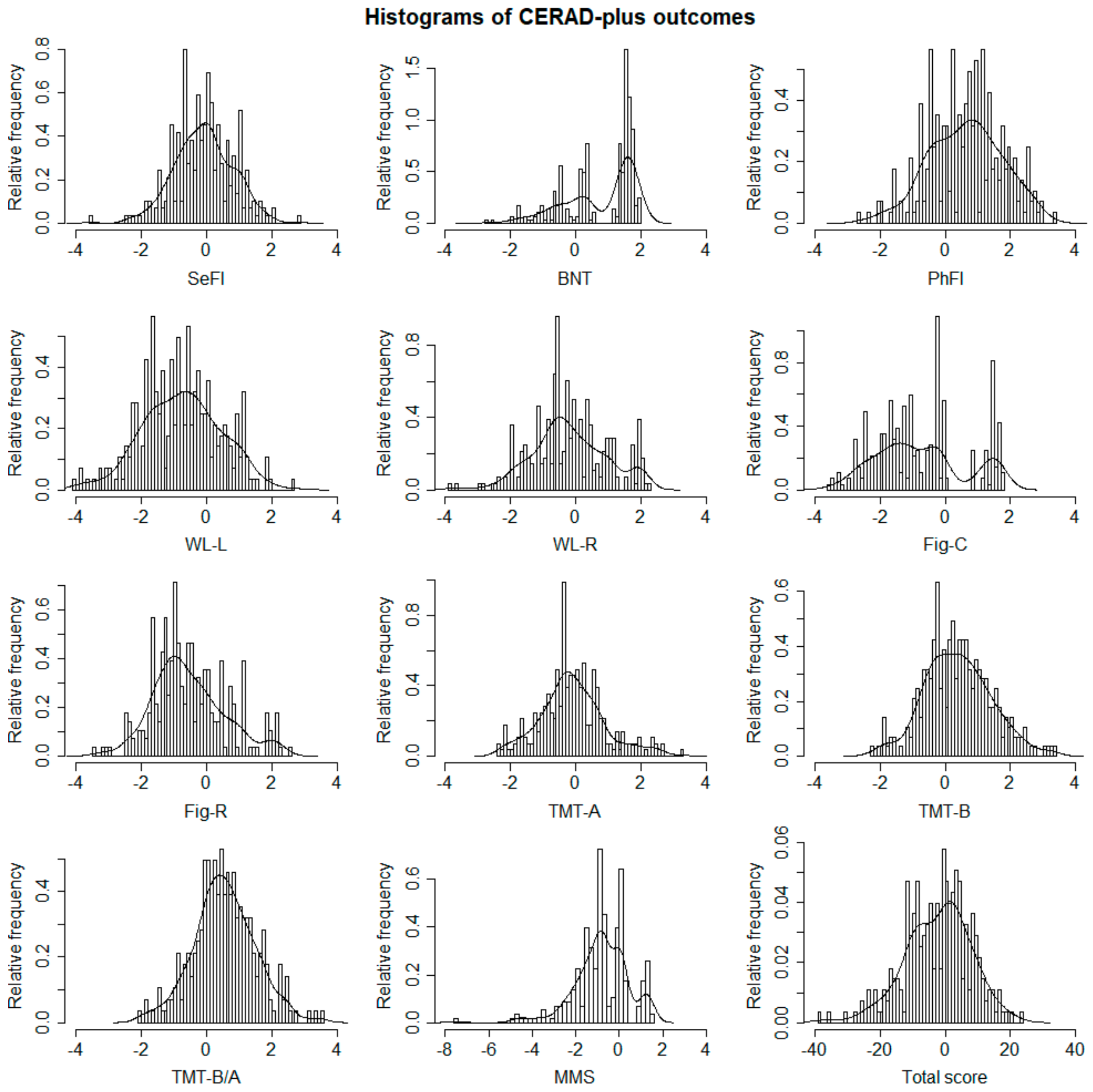

Figure S1. Distributions of CERAD-Plus z-scores in main sample ($N = 288$).
